# Supplementary material for: Use of DNA Barcoding Combined with PCR-SFLP to Authenticate Species in Bison Meat Products
Source: Foods. 2021 Feb 6;10(2):347. doi: 10.3390/foods10020347 (PMC7915063; doi:10.3390/foods10020347)
Supplement: Supplementary file 1 [file foods-10-00347-s001.pdf]

foods MDPI

DNA barcode sequence data for bison products tested in this study.

>Z001_2
TACCCTTTATCTACTATTTGGAGCCTGGGCCGGTATAGTAGGAACAGCTCTAAGCCTTCTAATTCGCGCTGAAT
TAGGCCAACCCGGAACCCTGCTCGGAGACGACCAAATCTACAACGTAGTTGTAACCGCACACGCATTTGTAA
TAATCTTCTTTATAGTAATGCCAATTATAATTGGAGGATTCGGTAACTGACTTGTTCCTCTAATAATTGGCGCT
CCCGACATGGCATTCCCCCGAATAAACAATATAAGCTTCTGACTCCTCCCTCCCTCATTTCTACTACTCCTCGC
ATCCTCTATAGTTGAAGCTGGAGCAGGAACAGGCTGAACCGTGTACCCTCCCTTAGCAGGCAACCTAGCCCA
TGCAGGAGCCTCAGTAGATCTAACCATCTTCTCCTTACACTTGGCAGGAGTTTCCTCTATTTTAGGAGCCATCA
ACTTCATCACAACAATTATCAACATAAAGCCCCCCGCAATGTCACAGTACCAAACCCCTCTCTTCGTATGATC
CGTAATAATTACCGCCGTACTATTACTCCTCTCACTTCCTGTGCTAGCAGCCGGTATTACAATGCTGCTAACAG
ATCGGAACCTAAATACAACCTTCTTCGACCCAGCAGGAGGAGGAGACCCCATCTTATACCAACACTTATTC
>Z002_2
TACCCTTTATCTACTATTTGGAGCCTGGGCCGGTATAGTAGGAACAGCTCTAAGCCTTCTAATTCGCGCTGAAT
TAGGCCAACCCGGAACCCTGCTCGGAGACGACCAAATCTACAACGTAGTTGTAACCGCACACGCATTTGTAA
TAATCTTCTTTATAGTAATGCCAATTATAATTGGAGGATTCGGTAACTGACTTGTTCCTCTAATAATTGGCGCT
CCCGACATGGCATTCCCCCGAATAAACAATATAAGCTTCTGACTCCTCCCTCCCTCATTTCTACTACTCCTCGC
ATCCTCTATAGTTGAAGCTGGAGCAGGAACAGGCTGAACCGTGTACCCTCCCTTAGCAGGCAACCTAGCCCA
TGCAGGAGCCTCAGTAGATCTAACCATCTTCTCCTTACACTTGGCAGGAGTTTCCTCTATTTTAGGAGCCATCA
ACTTCATCACAACAATTATCAACATAAAGCCCCCCGCAATATCACAGTACCAAACCCCTCTCTTCGTATGATC
CGTAATAATTACCGCCGTACTATTACTCCTCTCACTTCCTGTGCTAGCAGCCGGTATTACAATGCTGCTAACAG
ATCGGAACCTAAATACAACCTTCTTCGACCCAGCAGGAGGAGGAGACCCCATCTTATACCAACACTTATTC
>Z003_2
TACCCTTTATCTACTATTTGGTGCTTGGGCCGGTATAGTAGGAACAGCTCTAAGCCTTCTAATTCGCGCTGAAT
TAGGCCAACCCGGAACTCTGCTCGGAGACGACCAAATCTACAACGTAGTTGTAACCGCACACGCATTTGTAA
TAATCTTCTTCATAGTAATACCAATCATAATTGGAGGATTCGGTAACTGACTTGTTCCCCTAATAATTGGTGCT
CCCGAYATAGCATTTCCCCGAATAAATAATATAAGCTTCTGACTCCTCCCTCCCTCATTCCTACTACTCCTCGC
ATCCTCTATAGTTGAAGCTGGGGCAGGAACAGGCTGAACCGTGTACCCTCCCTTAGCAGGCAACCTAGCCCA
TGCAGGAGCTTCAGTAGATCTAACCATTTTCTCTTTACACTTAGCAGGAGTTTCCTCAATTTTAGGAGCCATCA
ACTTCATTACAACAATTATCAACATAAAGCCCCCCGCAATGTCACAATACCAAACCCCTCTGTTCGTATGATC
CGTAATAATTACCGCCGTACTAYTACTMCTCTCGCTTCCTGTATTAGCAGCCGGTATCACAATGCTATTAACA
GACCGGAACCTAAATACAACCTTCTTCGACCCRGCAGGAGGAGGAGACCCCATTCTATATCAACACTTATTC
>Z004_2
TACCCTTTATCTACTATTTGGAGCCTGGGCCGGTATAGTAGGAACAGCTCTAAGCCTTCTAATTCGCGCTGAAT
TAGGCCAACCCGGAACCCTGCTCGGAGACGACCAAATCTACAACGTAGTTGTAACCGCACACGCATTTGTAA
TAATCTTCTTTATAGTAATGCCAATTATAATTGGAGGATTCGGTAACTGACTTGTTCCTCTAATAATTGGCGCT
CCCGACATGGCATTCCCCCGAATAAACAATATAAGCTTCTGACTCCTCCCTCCCTCATTTCTACTACTCCTCGC
ATCCTCTATAGTTGAAGCTGGAGCAGGAACAGGCTGAACCGTGTACCCTCCCTTAGCAGGCAACCTAGCCCA
TGCAGGAGCCTCAGTAGATCTAACCATCTTCTCCTTACACTTGGCAGGAGTTTCCTCTATTTTAGGAGCCATCA
ACTTCATCACAACAATTATCAACATAAAGCCCCCCGCAATGTCACAGTACCAAACCCCTCTCTTCGTATGATC
CGTAATAATTACCGCCGTACTATTACTCCTCTCACTTCCTGTGCTAGCAGCCGGTATTACAATGCTGCTAACAG
ATCGGAACCTAAATACAACCTTCTTCGACCCAGCAGGAGGAGGAGACCCCATCTTATACCAACACTTATTC

>Z005_2
TACCCTTTATCTACTATTTGGAGCCTGGGCCGGTATAGTAGGAACAGCTCTAAGCCTTCTAATTCGCGCTGAAT
TAGGCCAACCCGGAACCCTGCTCGGAGACGACCAAATCTACAACGTAGTTGTAACCGCACACGCATTTGTAA
TAATCTTCTTTATAGTAATGCCAATTATAATTGGAGGATTCGGTAACTGACTTGTTCCTCTAATAATTGGCGCT
CCCGACATGGCATTCCCCCGAATAAACAATATAAGCTTCTGACTCCTCCCTCCCTCATTTCTACTACTCCTCGC
ATCCTCTATAGTTGAAGCTGGAGCAGGAACAGGCTGAACCGTGTACCCTCCCTTAGCAGGCAACCTAGCCCA
TGCAGGAGCCTCAGTAGATCTAACCATCTTCTCCTTACACTTGGCAGGAGTTTCCTCTATTTTAGGAGCCATCA
ACTTCATCACAACAATTATCAACATAAAGCCCCCCGCAATATCACAGTACCAAACCCCTCTCTTCGTATGATC
CGTAATAATTACCGCCGTACTATTACTCCTCTCACTTCCTGTGCTAGCAGCCGGTATTACAATGCTGCTAACAG
ATCGGAACCTAAATACAACCTTCTTCGACCCAGCAGGAGGAGGAGACCCCATCTTATACCAACA
>Z006_2
TACCCTTTATCTACTATTTGGAGCCTGGGCCGGTATAGTAGGAACAGCTCTAAGCCTTCTAATTCGCGCTGAAT
TAGGCCAACCCGGAACCCTGCTCGGAGACGACCAAATCTACAACGTAGTTGTAACCGCACACGCATTTGTAA
TAATCTTCTTTATAGTAATGCCAATTATAATTGGAGGATTCGGTAACTGACTTGTTCCTCTAATAATTGGCGCT
CCCGACATGGCATTCCCCCGAATAAACAATATAAGCTTCTGACTCCTCCCTCCCTCATTTCTACTACTCCTCGC
ATCCTCTATAGTTGAAGCTGGAGCAGGAACAGGCTGAACCGTGTACCCTCCCTTAGCAGGCAACCTAGCCCA
TGCAGGAGCCTCAGTAGATCTAACCATCTTCTCCTTACACTTGGCAGGAGTTTCCTCTATTTTAGGAGCCATCA
ACTTCATCACAACAATTATCAACATAAAGCCCCCCGCAATATCACAGTACCAAACCCCTCTCTTCGTATGATC
CGTAATAATTACCGCCGTACTATTACTCCTCTCACTTCCTGTGCTAGCAGCCGGTATTACAATGCTGCTAACAG
ATCGGAACCTAAATACAACCTTCTTCGACCCAGCAGGAGGAGGAGACCCCATCTTATACCAACACTTATTC
>Z007_2
TCTACTATTTGGAGCCTGGGCCGGTATAGTAGGAACAGCTCTAAGCCTTCTAATTCGCGCTGAATTAGGCCAA
CCCGGAACCCTGCTCGGAGACGACCAAATCTACAACGTAGTTGTAACCGCACACGCATTTGTAATAATCTTCT
TTATAGTAATGCCAATTATAATTGGAGGATTCGGTAACTGACTTGTTCCTCTAATAATTGGCGCTCCCGACATG
GCATTCCCCCGAATAAACAATATAAGCTTCTGACTCCTCCCTCCCTCATTTCTACTACTCCTCGCATCCTCTAT
AGTTGAAGCTGGAGCAGGAACAGGCTGAACCGTGTACCCTCCCTTAGCAGGCAACCTAGCCCATGCAGGAG
CCTCAGTAGATCTAACCATCTTCTCCTTACACTTGGCAGGAGTTTCCTCTATTTTAGGAGCCATCAACTTCATC
ACAACAATTATCAACATAAAGCCCCCCGCAATGTCACAGTACCAAACCCCTCTCTTCGTATGATCCGTAATA
ATTACCGCCGTACTATTACTCCTCTCACTTCCTGTGCTAGCAGCCGGTATTACAATGCTGCTAACAGATCGGA
ACCTAAATACAACCTTCTTCGACCCAGCAGGAGGAGGAGACCCCATCTTATACCAACACTTATTC
>Z008_2
CTACTATTTGGAGCCTGGGCCGGTATAGTAGGAACAGCTCTAAGCCTTCTAATTCGCGCTGAATTAGGCCAAC
CCGGAACCCTGCTCGGAGACGACCAAATCTACAACGTAGTTGTAACCGCACACGCATTTGTAATAATCTTCTT
TATAGTAATGCCAATTATAATTGGAGGATTCGGTAACTGACTTGTTCCTCTAATAATTGGCGCTCCCGACATG
GCATTCCCCCGAATAAACAATATAAGCTTCTGACTCCTCCCTCCCTCATTTCTACTACTCCTCGCATCCTCTAT
AGTTGAAGCTGGAGCAGGAACAGGCTGAACCGTGTACCCTCCCTTAGCAGGCAACCTAGCCCATGCAGGAG
CCTCAGTAGATCTAACCATCTTCTCCTTACACTTGGCAGGAGTTTCCTCTATTTTAGGAGCCATCAACTTCATC
ACAACAATTATCAACATAAAGCCCCCCGCAATATCACAGTACCAAACCCCTCTCTTCGTATGATCCGTAATA
ATTACCGCCGTACTATTACTCCTCTCACTTCCTGTGCTAGCAGCCGGTATTACAATGCTGCTAACAGATCGGA
ACCTAAATACAACCTTCTTCGACCCAGCAGGAGGAGGAGACCCCATCTTATACCAACACTTATTCT
>Z009_2

TACCCTTTATCTACTATTTGGAGCCTGGGCCGGTATAGTAGGAACAGCTCTAAGCCTTCTAATTCGCGCTGAAT
TAGGCCAACCCGGAACCCTGCTCGGAGACGACCAAATCTACAACGTAGTTGTAACCGCACACGCATTTGTAA
TAATCTTCTTTATAGTAATGCCAATTATAATTGGAGGATTCGGTAACTGACTTGTTCCTCTAATAATTGGCGCT
CCCGACATGGCATTCCCCCGAATAAACAATATAAGCTTCTGACTCCTCCCTCCCTCATTTCTACTACTCCTCGC
ATCCTCTATAGTTGAAGCTGGAGCAGGAACAGGCTGAACCGTGTACCCTCCCTTAGCAGGCAACCTAGCCCA
TGCAGGAGCCTCAGTAGATCTAACCATCTTCTCCTTACACTTGGCAGGAGTTTCCTCTATTTTAGGAGCCATCA
ACTTCATCACAACAATTATCAACATAAAGCCCCCCGCAATGTCACAGTACCAAACCCCTCTCTTCGTATGATC
CGTAATAATTACCGCCGTACTATTACTCCTCTCACTTCCTGTGCTAGCAGCCGGTATTACAATGCTGCTAACAG
ATCGGAACCTAAATACAACCTTCTTCGACCCAGCAGGAGGAGGAGACCCCATCTTATACCAACACTTATTC
>Z010_2
TACCCTTTATCTACTATTTGGAGCCTGGGCCGGTATAGTAGGAACAGCTCTAAGCCTTCTAATTCGCGCTGAAT
TAGGCCAACCCGGAACCCTGCTCGGAGACGACCAAATCTACAACGTAGTTGTAACCGCACACGCATTTGTAA
TAATCTTCTTTATAGTAATGCCAATTATAATTGGAGGATTCGGTAACTGACTTGTTCCTCTAATAATTGGCGCT
CCCGACATGGCATTCCCCCGAATAAACAATATAAGCTTCTGACTCCTCCCTCCCTCATTTCTACTACTCCTCGC
ATCCTCTATAGTTGAAGCTGGAGCAGGAACAGGCTGAACCGTGTACCCTCCCTTAGCAGGCAACCTAGCCCA
TGCAGGAGCCTCAGTAGATCTAACCATCTTCTCCTTACACTTGGCAGGAGTTTCCTCTATTTTAGGAGCCATCA
ACTTCATCACAACAATTATCAACATAAAGCCCCCCGCAATGTCACAGTACCAAACCCCTCTCTTCGTATGATC
CGTAATAATTACCGCCGTACTATTACTCCTCTCACTTCCTGTGCTAGCAGCCGGTATTACAATGCTGCTAACAG
ATCGGAACCTAAATACAACCTTCTTCGACCCAGCAGGAGGAGGAGACCCCATCTTATACCAACACTTATTC
>Z011_2
TACCCTTTATCTACTATTTGGTGCTTGGGCCGGTATAGTAGGAACAGCTCTAAGCCTTCTAATTCGCGCTGAAT
TAGGCCAACCCGGAACTCTGCTCGGAGACGACCAAATCTACAACGTAGTTGTAACCGCACACGCATTTGTAA
TAATCTTCTTCATAGTAATACCAATCATAATTGGAGGATTCGGTAACTGACTTGTTCCCCTAATAATTGGTGCT
CCCGATATAGCATTTCCCCGAATAAATAATATAAGCTTCTGACTCCTCCCTCCCTCATTCCTACTACTCCTCGC
ATCCTCTATAGTTGAAGCTGGGGCAGGAACAGGCTGAACCGTGTACCCTCCCTTAGCAGGCAACCTAGCCCA
TGCAGGAGCTTCAGTAGATCTAACCATTTTCTCTTTACACTTAGCAGGAGTTTCCTCAATTTTAGGAGCCATCA
ACTTCATTACAACAATTATCAACATAAAGCCCCCCGCAATGTCACAATACCAAACCCCTCTGTTCGTATGATC
CGTAATAATTACCGCCGTACTACTACTACTCTCGCTCCCTGTATTAGCAGCCGGCATCACAATGCTATTAACA
GACCGGAACCTAAATACAACCTTCTTCGACCCGGCAGGAGGAGGAGACCCTATTCTATATCAACACTTATTC
>Z012_2
TACCCTTTATCTACTATTTGGAGCCTGGGCCGGTATAGTAGGAACAGCTCTAAGCCTTCTAATTCGCGCTGAAT
TAGGCCAACCCGGAACCCTGCTCGGAGACGACCAAATCTACAACGTAGTTGTAACCGCACACGCATTTGTAA
TAATCTTCTTTATAGTAATGCCAATTATAATTGGAGGATTCGGTAACTGACTTGTTCCTCTAATAATTGGCGCT
CCCGACATGGCATTCCCCCGAATAAACAATATAAGCTTCTGACTCCTCCCTCCCTCATTTCTACTACTCCTCGC
ATCCTCTATAGTTGAAGCTGGAGCAGGAACAGGCTGAACCGTGTACCCTCCCTTAGCAGGCAACCTAGCCCA
TGCAGGAGCCTCAGTAGATCTAACCATCTTCTCCTTACACTTGGCAGGAGTTTCCTCTATTTTAGGAGCCATCA
ACTTCATCACAACAATTATCAACATAAAGCCCCCCGCAATGTCACAGTACCAAACCCCTCTCTTCGTATGATC
CGTAATAATTACCGCCGTACTATTACTCCTCTCACTTCCTGTGCTAGCAGCCGGTATTACAATGCTGCTAACAG
ATCGGAACCTAAATACAACCTTCTTCGACCCAGCAGGAGGAGGAGACCCCATCTTATACCAACACTTATTC
>Z013_2
TACCCTTTATCTACTATTTGGAGCCTGGGCCGGTATAGTAGGAACAGCTCTAAGCCTTCTAATTCGCGCTGAAT
TAGGCCAACCCGGAACCCTGCTCGGAGACGACCAAATCTACAACGTAGTTGTAACCGCACACGCATTTGTAA

TAATCTTCTTTATAGTAATGCCAATTATAATTGGAGGATTCGGTAACTGACTTGTTCCTCTAATAATTGGCGCT
CCCGACATGGCATTCCCCCGAATAAACAATATAAGCTTCTGACTCCTCCCTCCCTCATTTCTACTACTCCTCGC
ATCCTCTATAGTTGAAGCTGGAGCAGGAACAGGCTGAACCGTGTACCCTCCCTTAGCAGGCAACCTAGCCCA
TGCAGGAGCCTCAGTAGATCTAACCATCTTCTCCTTACACTTGGCAGGAGTTTCCTCTATTTTAGGAGCCATCA
ACTTCATCACAACAATTATCAACATAAAGCCCCCCGCAATATCACAGTACCAAACCCCTCTCTTCGTATGATC
CGTAATAATTACCGCCGTACTATTACTCCTCTCACTTCCTGTGCTAGCAGCCGGTATTACAATGCTGCTAACAG
ATCGGAACCTAAATACAACCTTCTTCGACCCAGCAGGAGGAGGAGACCCCATCTTATACCAACACTTATTC
>Z014_2
TACCCTTTATCTACTATTTGGTGCTTGGGCCGGTATAGTAGGAACAGCTCTAAGCCTTCTAATTCGCGCTGAAT
TAGGCCAACCCGGAACTCTGCTCGGAGACGACCAAATCTACAACGTAGTTGTAACCGCACACGCATTTGTAA
TAATCTTCTTCATAGTAATACCAATCATAATTGGAGGATTCGGTAACTGACTTGTTCCCCTAATAATTGGTGCT
CCCGATATAGCATTTCCCCGAATAAATAATATAAGCTTCTGACTCCTCCCTCCCTCATTCCTACTACTCCTCGC
ATCCTCTATAGTTGAAGCTGGGGCAGGAACAGGCTGAACCGTGTACCCTCCCTTAGCAGGCAACCTAGCCCA
TGCAGGAGCTTCAGTAGATCTAACCATTTTCTCTTTACACTTAGCAGGAGTTTCCTCAATTTTAGGAGCCATCA
ACTTCATTACAACAATTATCAACATAAAGCCCC
CCGCAATGTCACAATACCAAACCCCTCTGTTCGTATGATCCGTAATAATTACCGCCGTACTACTACTACTCTC
GCTCCCTGTATTAGCAGCCGGCATCACAATGCTATTAACAGACCGGAACCTAAATACAACCTTCTTCGACCCG
GCAGGAGGAGGAGACCCTATTCTATATCAACACTTATTC
>Z015_2
TACCCTTTATCTACTATTTGGAGCCTGGGCCGGTATAGTAGGAACAGCTCTAAGCCTTCTAATTCGCGCTGAAT
TAGGCCAACCCGGAACCCTGCTCGGAGACGACCAAATCTACAACGTAGTTGTAACCGCACACGCATTTGTAA
TAATCTTCTTTATAGTAATGCCAATTATAATTGGAGGATTCGGTAACTGACTTGTTCCTCTAATAATTGGCGCT
CCCGACATGGCATTCCCCCGAATAAACAATATAAGCTTCTGACTCCTCCCTCCCTCATTTCTACTACTCCTCGC
ATCCTCTATAGTTGAAGCTGGAGCAGGAACAGGCTGAACCGTGTACCCTCCCTTAGCAGGCAACCTAGCCCA
TGCAGGAGCCTCAGTAGATCTAACCATCTTCTCCTTACACTTGGCAGGAGTTTCCTCTATTTTAGGAGCCATCA
ACTTCATCACAACAATTATCAACATAAAGCCCCCCGCAATGTCACAGTACCAAACCCCTCTCTTCGTATGATC
CGTAATAATTACCGCCGTACTATTACTCCTCTCACTTCCTGTGCTAGCAGCCGGTATTACAATGCTGCTAACAG
ATCGGAACCTAAATACAACCTTCTTCGACCCAGCAGGAGGAGGAGACCCCATCTTATACCAACACTTATTC
>Z016_2
TACCCTTTATCTACTATTTGGAGCCTGGGCCGGTATAGTAGGAACAGCTCTAAGCCTTCTAATTCGCGCTGAAT
TAGGCCAACCCGGAACCCTGCTCGGAGACGACCAAATCTACAACGTAGTTGTAACCGCACACGCATTTGTAA
TAATCTTCTTTATAGTAATGCCAATTATAATTGGAGGATTCGGTAACTGACTTGTTCCTCTAATAATTGGCGCT
CCCGACATGGCATTCCCCCGAATAAACAATATAAGCTTCTGACTCCTCCCTCCCTCATTTCTACTACTCCTCGC
ATCCTCTATAGTTGAAGCTGGAGCAGGAACAGGCTGAACCGTGTACCCTCCCTTAGCAGGCAACCTAGCCCA
TGCAGGAGCCTCAGTAGATCTAACCATCTTCTCCTTACACTTGGCAGGAGTTTCCTCTATTTTAGGAGCCATCA
ACTTCATCACAACAATTATCAACATAAAGCCCCCCGCAATGTCACAGTACCAAACCCCTCTCTTCGTATGATC
CGTAATAATTACCGCCGTACTATTACTCCTCTCACTTCCTGTGCTAGCAGCCGGTATTACAATGCTGCTAACAG
ATCGGAACCTAAATACAACCTTCTTCGACCCAGCAGGAGGAGGAGACCCCATCTTATACCAACACTTATTC
>Z017_2
TACCCTTTATCTACTATTTGGAGCCTGGGCCGGTATAGTAGGAACAGCTCTAAGCCTTCTAATTCGCGCTGAAT
TAGGCCAACCCGGAACCCTGCTCGGAGACGACCAAATCTACAACGTAGTTGTAACCGCACACGCATTTGTAA
TAATCTTCTTTATAGTAATGCCAATTATAATTGGAGGATTCGGTAACTGACTTGTTCCTCTAATAATTGGCGCT

CCCGACATGGCATTCCCCCGAATAAACAATATAAGCTTCTGACTCCTCCCTCCCTCATTTCTACTACTCCTCGC
ATCCTCTATAGTTGAAGCTGGAGCAGGAACAGGCTGAACCGTGTACCCTCCCTTAGCAGGCAACCTAGCCCA
TGCAGGAGCCTCAGTAGATCTAACCATCTTCTCCTTACACTTGGCAGGAGTTTCCTCTATTTTAGGAGCCATCA
ACTTCATCACAACAATTATCAACATAAAGCCCCCCGCAATRTCACAGTACCAAACCCCTCTCTTCGTATGATC
CGTAATAATTACCGCCGTACTATTACTCCTCTCACTTCCTGTGCTAGCAGCCGGTATTACAATGCTGCTAACAG
ATCGGAACCTAAATACAACCTTCTTCGACCCAGCAGGAGGAGGAGACCCCATCTTATACCAACACTCTATTC
>Z018_2
TACCCTTTATCTACTATTTGGAGCCTGGGCCGGTATAGTAGGAACAGCTCTAAGCCTTCTAATTCGCGCTGAAT
TAGGCCAACCCGGAACCCTGCTCGGAGACGACCAAATCTACAACGTAGTTGTAACCGCACACGCATTTGTAA
TAATCTTCTTTATAGTAATGCCAATTATAATTGGAGGATTCGGTAACTGACTTGTTCCTCTAATAATTGGCGCT
CCCGACATGGCATTCCCCCGAATAAACAATATAAGCTTCTGACTCCTCCCTCCCTCATTTCTACTACTCCTCGC
ATCCTCTATAGTTGAAGCTGGAGCAGGAACAGGCTGAACCGTGTACCCTCCCTTAGCAGGCAACCTAGCCCA
TGCAGGAGCCTCAGTAGATCTAACCATCTTCTCCTTACACTTGGCAGGAGTTTCCTCTATTTTAGGAGCCATCA
ACTTCATCACAACAATTATCAACATAAAGCCCCCCGCAATGTCACAGTACCAAACCCCTCTCTTCGTATGATC
CGTAATAATTACCGCCGTACTATTACTCCTCTCACTTCCTGTGCTAGCAGCCGGTATTACAATGCTGCTAACAG
ATCGGAACCTAAATACAACCTTCTTCGACCCAGCAGGAGGAGGAGACCCCATCTTATATCAACACTTATTC
>Z019_2
TACCCTTTATCTACTATTTGGAGCCTGGGCCGGTATAGTAGGAACAGCTCTAAGCCTTCTAATTCGCGCTGAAT
TAGGCCAACCCGGAACCCTGCTCGGAGACGACCAAATCTACAACGTAGTTGTAACCGCACACGCATTTGTAA
TAATCTTCTTTATAGTAATGCCAATTATAATTGGAGGATTCGGTAACTGACTTGTTCCTCTAATAATTGGCGCT
CCCGACATGGCATTCCCCCGAATAAACAATATAAGCTTCTGACTCCTCCCTCCCTCATTTCTACTACTCCTCGC
ATCCTCTATAGTTGAAGCTGGAGCAGGAACAGGCTGAACCGTGTACCCTCCCTTAGCAGGCAACCTAGCCCA
TGCAGGAGCCTCAGTAGATCTAACCATCTTCTCCTTACACTTGGCAGGAGTTTCCTCTATTTTAGGAGCCATCA
ACTTCATCACAACAATTATCAACATAAAGCCCCCCGCAATGTCACAGTACCAAACCCCTCTCTTCGTATGATC
CGTAATAATTACCGCCGTACTATTACTCCTCTCACTTCCTGTGCTAGCAGCCGGTATTACAATGCTGCTAACAG
ATCGGAACCTAAATACAACCTTCTTCGACCCAGCAGGAGGAGGAGACCCCATCTTATATCAACACTTATTC
>Z020_2
CTACTATTTGGAGCCTGGGCCGGTATAGTAGGAACAGCTCTAAGCCTTCTAATTCGCGCTGAATTAGGCCAAC
CCGGAACCCTGCTCGGAGACGACCAAATCTACAACGTAGTTGTAANCCGCACACGCATTTGTAATAWTCTTC
TTTATAGTAATGCCAATTATAANTKGGAGGATTCGGTAACTGACTTGTTCCTCTAATAATTGGCGCTCCCGAC
ATGGCATTCCCCCGAAATAAAACAATATAAGCTTCTGACTCCTCCCTCCCTCATTTNCTACTACTCCTCGCATC
CTCTATAGTTTGAAGCTGGAGCAGGAACAGGCTGAACCGTGTACCCTCCCTTAGCAGGCAACCTAGCCCATG
CAGGAGCCTCAGTAGATCTAACCATCTTCTCCTTACACTTGGCAGGAGTTTCCTCTATTTTAGGAGCCATCAAC
TTCATCACAACAATTATCAACATAAAGCCCCCCGCAATGTCACAGTACCAAACCCCTCTCTTCGTATGATCCG
TAATAATTACCGCCGTACTATTACTCCTCTCACTTCCTGTGCTAGCAGCCGGTATTACAATGCTGCTAACAGAT
CGGAACCTAAATACAACCTTCTTCGACCCAGCAGGAGGAGGAGACCCC
>Z021_2
TACTCTGTATCTATTATTTGGTGCCTGAGCAGGCATAGTAGGGACAGCCTTAAGCCTACTGATTCGTGCCGAA
CTGGGCCAACCTGGTACTCTACTTGGAGATGACCAAATTTATAATGTTATCGTAACCGCACATGCATTCGTAA
TAATTTTCTTTATAGTTATGCCAATTATAATTGGAGGATTTGGTAATTGACTAGTTCCCCTAATAATTGGCGCC
CCAGACATAGCATTTCCTCGAATAAACAATATAAGCTTTTGACTCCTCCCTCCTTCTTTCTTACTACTCCTAGC
ATCATCTATAGTTGAAGCTGGCGCAGGAACAGGCTGAACTGTATATCCCCCTCTAGCTGGCAACTTAGCTCAT

GCAGGGGCTTCAGTAGACCTGACTATTTTTCTTTACACTTAGCAGGCGTCTCCTCAATTCTAGGGGCCATTAA
CTTTATTACAACAATTATCAATATAAAACCCCCTGCCATATCACAATATCAAACCCCTCTATTTGTGTGATCCG
TATTAGTCACTGCTGTACTACTACTTCTCTCACTCCCTGTACTAGCAGCCGGAATTACAATACTATTAACAGAC
CGAAACTTAAATACAACCTTCTTTGACCCAGCAGGAGGCGGAGATCCTATTCTATATCAACACTTGTTC
>Z022_2
TACCCTTTATCTACTATTTGGAGCCTGGGCCGGTATAGTAGGAACAGCTCTAAGCCTTCTAATTCGCGCTGAAT
TAGGCCAACCCGGAACCCTGCTCGGAGACGACCAAATCTACAACGTAGTTGTAACCGCACACGCATTTGTAA
TAATCTTCTTTATAGTAATGCCAATTATAATTGGAGGATTCGGTAACTGACTTGTTCCTCTAATAATTGGCGCT
CCCGACATGGCATTCCCCCGAATAAACAATATAAGCTTCTGACTCCTCCCTCCCTCATTTCTACTACTCCTCGC
ATCCTCTATAGTTGAAGCTGGAGCAGGAACAGGCTGAACCGTGTACCCTCCCTTAGCAGGCAACCTAGCCCA
TGCAGGAGCCTCAGTAGATCTAACCATCTTCTCCTTACACTTGGCAGGAGTTTCCTCTATTTTAGGAGCCATCA
ACTTCATCACAACAATTATCAACATAAAGCCCCCCGCAATGTCACAGTACCAAACCCCTCTCTTCGTATGATC
CGTAATAATTACCGCCGTACTATTACTCCTCTCACTTCCTGTGCTAGCAGCCGGTATTACAATGCTGCTAACAG
ATCGGAACCTAAATACAACCTTCTTCGACCCAGCAGGAGGAGGAGACCCCATCTTATACCAACACTTATTC
>Z023_2
TACCCTTTATCTACTATTTGGAGCCTGGGCCGGTATAGTAGGAACAGCTCTAAGCCTTCTAATTCGCGCTGAAT
TAGGCCAACCCGGAACCCTGCTCGGAGACGACCAAATCTACAACGTAGTTGTAACCGCACACGCATTTGTAA
TAATCTTCTTTATAGTAATGCCAATTATAATTGGAGGATTCGGTAACTGACTTGTTCCTCTAATAATTGGCGCT
CCCGACATGGCATTCCCCCGAATAAACAATATAAGCTTCTGACTCCTCCCTCCCTCATTTCTACTACTCCTCGC
ATCCTCTATAGTTGAAGCTGGAGCAGGAACAGGCTGAACCGTGTACCCTCCCTTAGCAGGCAACCTAGCCCA
TGCAGGAGCCTCAGTAGATCTAACCATCTTCTCCTTACACTTGGCAGGAGTTTCCTCTATTTTAGGAGCCATCA
ACTTCATCACAACAATTATCAACATAAAGCCCCCCGCAATGTCACAGTACCAAACCCCTCTCTTCGTATGATC
CGTAATAATTACCGCCGTACTATTACTCCTCTCACTTCCTGTGCTAGCAGCCGGTATTACAATGCTGCTAACAG
ATCGGAACCTAAATACAACCTTCTTCGACCCAGCAGGAGGAGGAGACCCCATCTTATACCAACACTTATTC
>Z024_2
TCTACTATTTGGAGCCTGGGCCGGTATAGTAGGAACAGCTCTAAGCCTTCTAATTCGCGCTGAATTAGGCCAA
CCCGGAACCCTGCTCGGAGACGACCAAATCTACAACGTAGTTGTAACCGCACACGCATTTGTAATAATCTTCT
TTATAGTAATGCCAATTATAATTGGAGGATTCGGTAACTGACTTGTTCCTCTAATAATTGGCGCTCCCGACATG
GCATTCCCCCGAATAAACAATATAAGCTTCTGACTCCTCCCTCCCTCATTTCTACTACTCCTCGCATCCTCTAT
AGTTGAAGCTGGAGCAGGAACAGGCTGAACCGTGTACCCTCCCTTAGCAGGCAACCTAGCCCCATGCAGGA
GCCTCAGTAGATCTAACCATCTTCTCCTTACACTTGGCAGGAGTTTCCTCTATTTTAGGAGCCATCAACTTCAT
CACAACAATTATCAACATAAAGCCCCCCGCAATGTCACAGTACCAAACCCCTCTCTTCGTATGATCCGTAATA
ATTACCGCCGTACTATTACTCCTCTCACTTCCTGTGCTAGCAGCCGGTATTACAATGCTGCTAACAGATCGGA
ACCTAAATACAACCTTCTTCGACCCAGCAGGAGGAGGAGACCCCATCTTATACCAACACTTATTC
>Z025_2
TACCCTTTATCTACTATTTGGAGCCTGGGCCGGTATAGTAGGAACAGCTCTAAGCCTTCTAATTCGCGCTGAAT
TAGGCCAACCCGGAACCCTGCTCGGAGACGACCAAATCTACAACGTAGTTGTAACCGCACACGCATTTGTAA
TAATCTTCTTTAT
AGTAATGCCAATTATAATTGGAGGATTCGGTAACTGACTTGTTCCTCTAATAATTGGCGCTCCCGACATGGCA
TTCCCCCGAATAAACAATATAAGCTTCTGACTCCTCCCTCCCTCATTTCTACTACTCCTCGCATCCTCTATAGTT
GAAGCTGGAGCAGGAACAGGCTGAACCGTGTACCCTCCCTTAGCAGGCAACCTAGCCCATGCAGGAGCCTC
AGTAGATCTAACCATCTTCTCCTTACACTTGGCAGGAGTTTCCTCTATTTTAGGAGCCATCAACTTCATCACAA

CAATTATCAACATAAAGCCCCCCGCAATATCACAGTACCAAACCCCTCTCTTCGTATGATCCGTAATAATTAC
CGCCGTACTATTACTCCTCTCACTTCCTGTGCTAGCAGCCGGTATTACAATGCTGCTAACAGATCGGAACCTA
AATACAACCTTCTTCGACCCAGCAGGAGGAGGAGACCCCATCTTATACCAACACTTATTC
>Z026_2
TACCCTTTATCTACTATTTGGAGCCTGGGCCGGTATAGTAGGAACAGCTCTAAGCCTTCTAATTCGCGCTGAAT
TAGGCCAACCCGGAACCCTGCTCGGAGACGACCAAATCTACAACGTAGTTGTAACCGCACACGCATTTGTAA
TAATCTTCTTTATAGTAATGCCAATTATAATTGGAGGATTCGGTAACTGACTTGTTCCTCTAATAATTGGCGCT
CCCGACATGGCATTCCCCCGAATAAACAATATAAGCTTCTGACTCCTCCCTCCCTCATTTCTACTACTCCTCGC
ATCCTCTATAGTTGAAGCTG
GAGCAGGAACAGGCTGAACCGTGTACCCTCCCTTAGCAGGCAACCTAGCCCATGCAGGAGCCTCAGTAGATC
TAACCATCTTCTCCTTACACTTGGCAGGAGTTTCCTCTATTTTAGGAGCCATCAACTTCATCACAACAATTATC
AACATAAAGCCCCCCGCAATGTCACAGTACCAAACCCCTCTCTTCGTATGATCCGTAATAATTACCGCCGTAC
TATTACTCCTCTCACTTCCTGTGCTAGCAGCCGGTATTACAATGCTGCTAACAGATCGGAACCTAAATACAAC
CTTCTTCGACCCAGCAGGAGGAGGAGACCCCATCTTATACCAACACTTATTC
>Z027_2
TACCCTTTATCTACTATTTGGAGCCTGGGCCGGTATAGTAGGAACAGCTCTAAGCCTTCTAATTCGCGCTGAAT
TAGGCCAACCCGGAACCCTGCTCGGAGACGACCAAATCTACAACGTAGTTGTAACCGCACACGCATTTGTAA
TAATCTTCTTTATAGTAATGCCAATTATAATTGGAGGATTCGGTAACTGACTTGTTCCTCTAATAATTGGCGCT
CCCGACATGGCATTCCCCCGAATAAACAATATAAGCTTCTGACTCCTCCCTCCCTCATTTCTACTACTCCTCGC
ATCCTCTATAGTTGAAGCTGGAGCAGGAACAGGCTGAACCGTGTACCCTCCCTTAGCAGGCAACCTAGCCCA
TGCAGGAGCCTCAGTAGATCTAACCATCTTCTCCTTACACTTGGCAGGAGTTTCCTCTATTTTAGGAGCCATCA
ACTTCATCACAACAATTATCAACATAAAGCCCCCCGCAATGTCACAGTACCAAACCCCTCTCTTCGTATGATC
CGTAATAATTACCGCCGTACTATTACTCCTCTCACTTCCTGTGCTAGCAGCCGGTATTACAATGCTGCTAACAG
ATCGGAACCTAAATACAACCTTCTTCGACCCAGCAGGAGGAGGAGACCCCATCTTATACCAACACTTATTC
>Z028_2
TACCCTTTATCTACTATTTGGAGCCTGGGCCGGTATAGTAGGAACAGCTCTAAGCCTTCTAATTCGCGCTGAAT
TAGGCCAACCCGGAACCCTGCTCGGAGACGACCAAATCTACAACGTAGTTGTAACCGCACACGCATTTGTAA
TAATCTTCTTTATAGTAATGCCAATTATAATTGGAGGATTCGGTAACTGACTTGTTCCTCTAATAATTGGCGCT
CCCGACATGGCATTCCCCCGAATAAACAATATAAGCTTCTGACTCCTCCCTCCCTCATTTCTACTACTCCTCGC
ATCCTCTATAGTTGAAGCTGGAGCAGGAACAGGCTGAACCGTGTACCCTCCCTTAGCAGGCAACCTAGCCCA
TGCAGGAGCCTCAGTAGATCTAACCATCTTCTCCTTACACTTGGCAGGAGTTTCCTCTATTTTAGGAGCCATCA
ACTTCATCACAACAATTATCAACATAAAGCCCCCCGCAATGTCACAGTACCAAACCCCTCTCTTCGTATGATC
CGTAATAATTACCGCCGTACTATTACTCCTCTCACTTCCTGTGCTAGCAGCCGGTATTACAATGCTGCTAACAG
ATCGGAACCTAAATACAACCTTCTTCGACCCAGCAGGAGGAGGAGACCCCATCTTATACCAACACTTATTC
>Z029_2
TACCCTTTATCTACTATTTGGAGCCTGGGCCGGTATAGTAGGAACAGCTCTAAGCCTTCTAATTCGCGCTGAAT
TAGGCCAACCCGGAACCCTGCTCGGAGACGACCAAATCTACAACGTAGTTGTAACCGCACACGCATTTGTAA
TAATCTTCTTTATAGTAATGCCAATTATAATTGGAGGATTCGGTAACTGACTTGTTCCTCTAATAATTGGCGCT
CCCGACATGGCATTCCCCCGAATAAACAATATAAGCTTCTGACTCCTCCCTCCCTCATTTCTACTACTCCTCGC
ATCCTCTATAGTTGAAGCTGGAGCAGGAACAGGCTGAACCGTGTACCCTCCCTTAGCAGGCAACCTAGCCCA
TGCAGGAGCCTCAGTAGATCTAACCATCTTCTCCTTACACTTGGCAGGAGTTTCCTCTATTTTAGGAGCCATCA
ACTTCATCACAACAATTATCAACATAAAGCCCCCCGCAATGTCACAGTACCAAACCCCTCTCTTCGTATGATC

CGTAATAATTACCGCCGTACTATTACTCCTCTCACTTCCTGTGCTAGCAGCCGGTATTACAATGCTGCTAACAG
ATCGGAACCTAAATACAACCTTCTTCGACCCAGCAGGAGGAGGAGACCCCATCTTATACCAACACTTATTC
>Z030_2
TACCCTTTATCTACTATTTGGAGCCTGGGCCGGTATAGTAGGAACAGCTCTAAGCCTTCTAATTCGCGCTGAAT
TAGGCCAACCCGGAACCCTGCTCGGAGACGACCAAATCTACAACGTAGTTGTAACCGCACACGCATTTGTAA
TAATCTTCTTTATAGTAATGCCAATTATAATTGGAGGATTCGGTAACTGACTTGTTCCTCTAATAATTGGCGCT
CCCGACATGGCATTCCCCCGAATAAACAATATAAGCTTCTGACTCCTCCCTCCCTCATTTCTACTACTCCTCGC
ATCCTCTATAGTTGAAGCTGGAGCAGGAACAGGCTGAACCGTGTACCCTCCCTTAGCAGGCAACCTAGCCCA
TGCAGGAGCCTCAGTAGATCTAACCATCTTCTCCTTACACTTGGCAGGAGTTTCCTCTATTTTAGGAGCCATCA
ACTTCATCACAACAATTATCAACATAAAGCCCCCCGCAATATCACAGTACCAAACCCCTCTCTTCGTATGATC
CGTAATAATTACCGCCGTACTATTACTCCTCTCACTTCCTGTGCTAGCAGCCGGTATTACAATGCTGCTAACAG
ATCGGAACCTAAATACAACCTTCTTCGACCCAGCAGGAGGAGGAGACCCCATCTTATACCAACACTTATTC
>Z031_2
TACTATTTGGAGCCTGGGCCGGTATAGTAGGAACAGCTCTAAGCCTTCTAATTCGCGCTGAATTAGGCCAACC
CGGAACCCTGCTCGGAGACGACCAAATCTACAACGTAGTTGTAACCGCACACGCATTTGTAATAATCTTCTTT
ATAGTAATGCCAATTATAATTGGAGGATTCGGTAACTGACTTGTTCCTCTAATAATTGGCGCTCCCGACATGG
CATTCCCCCGAATAAACAATATAAGCTTCTGACTCCTCCCTCCCTCATTTCTACTACTCCTCGCATCCTCTATA
GTTGAAGCTGGAGCAGGAACAGGCTGAACCGTGTACCCTCCCTTAGCAGGCAACCTAGCCCATGCAGGAGC
CTCAGTAGATCTAACCATCTTCTCCTTACACTTGGCAGGAGTTTCCTCTATTTTAGGAGCCATCAACTTCATCA
CAACAATTATCAACATAAAGCCCCCCGCAATRTCACAGTACCAAACCCCTCTCTTCGTATGATCCGTAATAAT
TACCGCCGTACTATTACTCCTCTCACTTCCTGTGCTAGCAGCCGGTATTACAATGCTGCTAACAGATCGGAAC
CTAAATACAACCTTCTTCGACCCAGCAGGAGGAGGAGACCCCATCTTATACCAACACTTATTC
>Z032_2
TACCCTTTATCTACTATTTGGAGCCTGGGCCGGTATAGTAGGAACAGCTCTAAGCCTTCTAATTCGCGCTGAAT
TAGGCCAACCCGGAACCCTGCTCGGAGACGACCAAATCTACAACGTAGTTGTAACCGCACACGCATTTGTAA
TAATCTTCTTTATAGTAATGCCAATTATAATTGGAGGATTCGGTAACTGACTTGTTCCTCTAATAATTGGCGCT
CCCGACATGGCATTCCCCCGAATAAACAATATAAGCTTCTGACTCCTCCCTCCCTCATTTCTACTACTCCTCGC
ATCCTCTATAGTTGAAGCTGGAGCAGGAACAGGCTGAACCGTGTACCCTCCCTTAGCAGGCAACCTAGCCCA
TGCAGGAGCCTCAGTAGATCTAACCATCTTCTCCTTACACTTGGCAGGAGTTTCCTCTATTTTAGGAGCCATCA
ACTTCATCACAACAATTATCAACATAAAGCCCCCCGCAATGTCACAGTACCAAACCCCTCTCTTCGTATGATC
CGTAATAATTACCGCCGTACTATTACTCCTCTCACTTCCTGTGCTAGCAGCCGGTATTACAATGCTGCTAACAG
ATCGGAACCTAAATACAACCTTCTTCGACCCAGCAGGAGGAGGAGACCCCATCTTATACCAACACTTATTC
>Z033_2
TACTATTTGGAGCCTGGGCCGGTATAGTAGGAACAGCTCTAAGCCTTCTAATTCGCGCTGAATTAGGCCAACC
CGGAACCCTGCTCGGAGACGACCAAATCTACACGTAGTTGTAACCGCACACGCATTTGTAATAATCTTCTTTA
TAGTAATGCCAATTATAATTGGAGGATTCGGTAACTGACTTGTTCCTCTAATAATTGGCGCTCCCGACATGGC
ATTCCCCCGAATAAACAATATAAGCTTCTGACTCCTCCCTCCCTCATTTCTACTACTCCTCGCATCCTCTATAG
TTGAAGCTGGAGCAGGAACAGGCTGAACCGTGTACCCTCCCTTAGCAGGCAACCTAGCCCATGCAGGAGCCT
CAGTAGATCTAACCATCTTCTCCTTACACTTGGCAGGAGTTTCCTCTATTTTAGGAGCCATCAACTTCATCACA
ACAATTATCAACATAAAGCCCCCCGCAATGTCACAGTACCAAACCCCTCTCTTCGTATGATCCGTAATAATTA
CCGCCGTACTATTACTCCTCTCACTTCCTGTGCTAGCAGCCGGTATTACAATGCTGCTAACAGATCGGAACCT
AAATACAACCTTCTTCGACCCAGCAGGAGGAGGAGACCCCATCTTATACCAACACTTATTC

>Z034_2
TACCCTTTATCTACTATTTGGAGCCTGGGCCGGTATAGTAGGAACAGCTCTAAGCCTTCTAATTCGCGCTGAAT
TAGGCCAACCCGGAACCCTGCTCGGAGACGACCAAATCTACAACGTAGTTGTAACCGCACACGCATTTGTAA
TAATCTTCTTTATAGTAATGCCAATTATAATTGGAGGATTCGGTAACTGACTTGTTCCTCTAATAATTGGCGCT
CCCGACATGGCATTCCCCCGAATAAACAATATAAGCTTCTGACTCCTCCCTCCCTCATTTCTACTACTCCTCGC
ATCCTCTATAGTTGAAGCTGGAGCAGGAACAGGCTGAACCGTGTACCCTCCCTTAGCAGGCAACCTAGCCCA
TGCAGGAGCCTCAGTAGATCTAACCATCTTCTCCTTACACTTGGCAGGAGTTTCCTCTATTTTAGGAGCCATCA
ACTTCATCACAACAATTATCAACATAAAGCCCCCCGCAATGTCACAGTACCAAACCCCTCTCTTCGTATGATC
CGTAATAATTACCGCCGTACTATTACTCCTCTCACTTCCTGTGCTAGCAGCCGGTATTACAATGCTGCTAACAG
ATCGGAACCTAAATACAACCTTCTTCGACCCAGCAGGAGGAGGAGACCCCATCTTATACCAACACTTATTC
>Z035_2
GGGCCGGTATAGTAGGAACAGCTCTAAGCCTTCTAATTCGCGCTGAATTAGGCCAACCCGGAACCCTGCTCG
GAGACGACCAAATCTACAACGTAGTTGTAACCGCACACGCATTTGTAATAATCTTCTTTATAGTAATGCCAAT
TATAATTGGAGGATTCGGTAACTGACTTGTTCCTCTAATAATTGGCGCTCCCGACATGGCATTCCCCCGAATA
AACAATATAAGCTTCTGACTCCTCCCTCCCTCATTTCTACTACTCCTCGCATCCTCTATAGTTGAAGCTGGAGC
AGGAACAGGCTGAACCGTGTACCCTCCCTTAGCAGGCAACCTAGCCCATGCAGGAGCCTCAGTAGATCTAAC
CATCTTCTCCTTACACTTGGCAGGAGTTTCCTCTATTTTAGGAGCCATCAACTTCATCACAACAATTATCAACA
TAAAGCCCCCCGCAATGTCACAGTACCAAACCCCTCTCTTCGTATGATCCGTAATAATTACCGCCGTACTATT
ACTCCTCTCACTTCCTGTGYTAGCAGCCGGTATTACAATGCTGCTAACAGATCGGAACCTAAATACAACCTTC
TTCGACCCAGCAGGAGGAGGAGACCCCATCTTATACCAACACTTATTC
>Z036_2
TACCCTTTATCTACTATTTGGAGCCTGGGCCGGTATAGTAGGAACAGCTCTAAGCCTTCTAATTCGCGCTGAAT
TAGGCCAACCCGGAACCCTGCTCGGAGACGACCAAATCTACAACGTAGTTGTAACCGCACACGCATTTGTAA
TAATCTTCTTTATAGTAATGCCAATTATAATTGGAGGATTCGGTAACTGACTTGTTCCTCTAATAATTGGCGCT
CCCGACATGGCATTCCCCCGAATAAACAATATAAGCTTCTGACTCCTCCCTCCCTCATTTCTACTACTCCTCGC
ATCCTCTATAGTTGAAGCTGGAGCAGGAACAGGCTGAACCGTGTACCCTCCCTTAGCAGGCAACCTAGCCCA
TGCAGGAGCCTCAGTAGATCTAACCATCTTCTCCTTACACTTGGCAGGAGTTTCCTCTATTTTAGGAGCCATCA
ACTTCATCACAACAATTATCAACATAAAGCCCCCCGCAATRTCACAGTACCAAACCCCTCTCTTCGTATGATC
CGTAATAATTACCGCCGTACTATTACTCCTCTCACTTCCTGTGCTAGCAGCCGGTATTACAATGCTGCTAACAG
ATCGGAACCTAAATACAACCTTCTTCGACCCAGCAGGAGGAGGAGACCCCATCTTATACCAACACTTATTC
>Z037_2
TACCCTTTATCTACTATTTGGAGCCTGGGCCGGTATAGTAGGAACAGCTCTAAGCCTTCTAATTCGCGCTGAAT
TAGGCCAACCCGGAACCCTGCTCGGAGACGACCAAATCTACAACGTAGTTGTAACCGCACACGCATTTGTAA
TAATCTTCTTTATAGTAATGCCAATTATAATTGGAGGATTCGGTAACTGACTTGTTCCTCTAATAATTGGCGCT
CCCGACATGGCATTCCCCCGAATAAACAATATAAGCTTCTGACTCCTCCCTCCCTCATTTCTACTACTCCTCGC
ATCCTCTATAGTTGAAGCTGGAGCAGGAACAGGCTGAACCGTGTACCCTCCCTTAGCAGGCAACCTAGCCCA
TGCAGGAGCCTCAGTAGATCTAACCATCTTCTCCTTACACTTGGCAGGAGTTTCCTCTATTTTAGGAGCCATCA
ACTTCATCACAACAATTATCAACATAAAGCCCCCCGCAATGTCACAGTACCAAACCCCTCTCTTCGTATGATC
CGTAATAATTACCGCCGTACTATTACTCCTCTCACTTCCTGTGCTAGCAGCCGGTATTACAATGCTGCTAACAG
ATCGGAACCTAAATACAACCTTCTTCGACCCAGCAGGAGGAGGAGACCCCATCTTATACCAACACTTATTC
>Z038_2

TCTACTATTKGGAGCCTGGGCCGGTATAGTAGGAACAGCTCTAAGCCTTCTAATTCGCGCTGAATTAGGCCAA
CCCGGAACCCTGCTCGGAGACGACCAAATCTACAACGTAGTTGTAACCGCACACGCATTTGTAATAATCTTCT
TTATAGTAATGCCAATTATAATTGGAGGATTCGGTAACTGACTTGTTCCTCTAATAATTGGCGCTCCCGACATG
GCATTCCCCCGAATAAACAATATAAGCTTCTGACTCCTCCCTCCCTCATTTCTACTACTCCTCGCATCCTCTAT
AGTTGAAGCTGGAGCAGGAACAGGCTGAACCGTGTACCCTCCCTTAGCAGGCAACCTAGCCCATGCAGGAG
CCTCAGTAGATCTAACCATCTTCTCCTTACACTTGGCAGGAGTTTCCTCTATTTTAGGAGCCATCAACTTCATC
ACAACAATTATCAACATAAAGCCCCCCGCAATATCACAGTACCAAACCCCTCTCTTCGTATGATCCGTAATA
ATTACCGCCGTACTATTACTCCTCTCACTTCCTGTGCTAGCAGCCGGTATTACAATGCTGCTAACAGATCGGA
ACCTAAATACAACCTTCTTCGACCCAGCAGGAGGAGGAGACCCCATCTTATACCAACACTTA
>Z039_2
TACCCTTTATCTACTATTTGGAGCCTGGGCCGGTATAGTAGGAACAGCTCTAAGCCTTCTAATTCGCGCTGAAT
TAGGCCAACCCGGAACCCTGCTCGGAGACGACCAAATCTACAACGTAGTTGTAACCGCACACGCATTTGTAA
TAATCTTCTTTATAGTAATGCCAATTATAATTGGAGGATTCGGTAACTGACTTGTTCCTCTAATAATTGGCGCT
CCCGACATGGCATTCCCCCGAATAAACAATATAAGCTTCTGACTCCTCCCTCCCTCATTTCTACTACTCCTCGC
ATCCTCTATAGTTGAAGCTGGAGCAGGAACAGGCTGAACCGTGTACCCTCCCTTAGCAGGCAACCTAGCCCA
TGCAGGAGCCTCAGTAGATCTAACCATCTTCTCCTTACACTTGGCAGGAGTTTCCTCTATTTTAGGAGCCATCA
ACTTCATCACAACAATTATCAACATAAAGCCCCCCGCAATGTCACAGTACCAAACCCCTCTCTTCGTATGATC
CGTAATAATTACCGCCGTACTATTACTCCTCTCACTTCCTGTGCTAGCAGCCGGTATTACAATGCTGCTAACAG
ATCGGAACCTAAATACAACCTTCTTCGACCCAGCAGGAGGAGGAGACCCCATCTTATACCAACACTTATTC
>Z040_2
TACCCTTTATCTACTATTTGGAGCCTGGGCCGGTATAGTAGGAACAGCTCTAAGCCTTCTAATTCGCGCTGAAT
TAGGCCAACCCGGAACCCTGCTCGGAGACGACCAAATCTACAACGTAGTTGTAACCGCACACGCATTTGTAA
TAATCTTCTTTATAGTAATGCCAATTATAATTGGAGGATTCGGTAACTGACTTGTTCCTCTAATAATTGGCGCT
CCCGACATGGCATTCCCCCGAATAAACAATATAAGCTTCTGACTCCTCCCTCCCTCATTTCTACTACTCCTCGC
ATCCTCTATAGTTGAAGCTGGGGCAGGAACAGGCTGAACCGTGTACCCTCCCTTAGCAGGCAACCTAGCCCA
TGCAGGAGCTTCAGTAGATCTAACCATCTTCTCCTTACACTTGGCAGGAGTTTCCTCTATTTTAGGAGCCATCA
ACTTCATCACAACAATTATCAACATAAAGCCCCCCGCAATGTCACARTACCAAACCCCTCTSTTCGTATGATC
CGTAATAATTACCGCCGTACTATTACTCCTCTCACTTCCTGTGCTAGCAGCCGGTATTACAATGCTGCTAACAG
ATCGGAACCTAAATACAACCTTCTTCGACCCAGCAGGAGGAGGAGACCCCATCTTATACCAACACTTATTC
>Z041_2
TACCCTTTATCTACTATTTGGAGCCTGGGCCGGTATAGTAGGAACAGCTCTAAGCCTTCTAATTCGCGCTGAAT
TAGGCCAACCCGGAACCCTGCTCGGAGACGACCAAATCTACAACGTAGTTGTAACCGCACACGCATTTGTAA
TAATCTTCTTTATAGTAATGCCAATTATAATTGGAGGATTCGGTAACTGACTTGTTCCTCTAATAATTGGCGCT
CCCGACATGGCATTCCCCCGAATAAACAATATAAGCTTCTGACTCCTCCCTCCCTCATTTCTACTACTCCTCGC
ATCCTCTATAGTTGAAGCTGGAGCAGGAACAGGCTGAACCGTGTACCCTCCCTTAGCAGGCAACCTAGCCCA
TGCAGGAGCCTCAGTAGATCTAACCATCTTCTCCTTACACTTGGCAGGAGTTTCCTCTATTTTAGGAGCCATCA
ACTTCATCACAACAATTATCAACATAAAGCCCCCCGCAATGTCACAGTACCAAACCCCTCTCTTCGTATGATC
CGTAATAATTACCGCCGTACTATTACTCCTCTCACTTCCTGTGCTAGCAGCCGGTATTACAATGCTGCTAACAG
ATCGGAACCTAAATACAACCTTCTTCGACCCAGCAGGAGGAGGAGACCCCATCTTATACCAACACTTATTC
>Z042_2
TACCCTTTATCTACTATTTGGAGCCTGGGCCGGTATAGTAGGAACAGCTCTAAGCCTTCTAATTCGCGCTGAAT
TAGGCCAACCCGGAACCCTGCTCGGAGACGACCAAATCTACAACGTAGTTGTAACCGCACACGCATTTGTAA

TAATCTTCTTTATAGTAATGCCAATTATAATTGGAGGATTCGGTAACTGACTTGTTCCTCTAATAATTGGCGCT
CCCGACATGGCATTCCCCCGAATAAACAATATAAGCTTCTGACTCCTCCCTCCCTCATTTCTACTACTCCTCGC
ATCCTCTATAGTTGAAGCTGGAGCAGGAACAGGCTGAACCGTGTACCCTCCCTTAGCAGGCAACCTAGCCCA
TGCAGGAGCCTCAGTAGATCTAACCATCTTCTCCTTACACTTGGCAGGAGTTTCCTCTATTTTAGGAGCCATCA
ACTTCATCACAACAATTATCAACATAAAGCCCCCCGCAATGTCACAGTACCAAACCCCTCTCTTCGTATGATC
CGTAATAATTACCGCCGTACTATTACTCCTCTCACTTCCTGTGCTAGCAGCCGGTATTACAATGCTGCTAACAG
ATCGGAACCTAAATACAACCTTCTTCGACCCAGCAGGAGGAGGAGACCCCATCTTATACCAACACTTATTC
>Z043_2
TACCCTTTATCTACTATTTGGAGCCTGGGCCGGTATAGTAGGAACAGCTCTAAGCCTTCTAATTCGCGCTGAAT
TAGGCCAACCCGGAACCCTGCTCGGAGACGACCAAATCTACAACGTAGTTGTAACCGCACACGCATTTGTAA
TAATCTTCTTTATAGTAATGCCAATTATAATTGGAGGATTCGGTAACTGACTTGTTCCTCTAATAATTGGCGCT
CCCGACATGGCATTCCCCCGAATAAACAATATAAGCTTCTGACTCCTCCCTCCCTCATTTCTACTACTCCTCGC
ATCCTCTATAGTTGAAGCTGGAGCAGGAACAGGCTGAACCGTGTACCCTCCCTTAGCAGGCAACCTAGCCCA
TGCAGGAGCCTCAGTAGATCTAACCATCTTCTCCTTACACTTGGCAGGAGTTTCCTCTATTTTAGGAGCCATCA
ACTTCATCACAACAATTATCAACATAAAGCCCCCCGCAATATCACAGTACCAAACCCCTCTCTTCGTATGATC
CGTAATAATTACCGCCGTACTATTACTCCTCTCACTTCCTGTGCTAGCAGCCGGTATTACAATGCTGCTAACAG
ATCGGAACCTAAATACAACCTTCTTCGACCCAGCAGGAGGAGGAGACCCCATCTTATACCAACACTTATTC
>Z044_2
TACCCTTTATCTACTATTTGGAGCCTGGGCCGGTATAGTAGGAACAGCTCTAAGCCTTCTAATTCGCGCTGAAT
TAGGCCAACCCGGAACCCTGCTCGGAGACGACCAAATCTACAACGTAGTTGTAACCGCACACGCATTTGTAA
TAATCTTCTTTATAGTAATGCCAATTATAATTGGAGGATTCGGTAACTGACTTGTTCCTCTAATAATTGGCGCT
CCCGACATGGCATTCCCCCGAATAAACAATATAAGCTTCTGACTCCTCCCTCCCTCATTTCTACTACTCCTCGC
ATCCTCTATAGTTGAAGCTGGAGCAGGAACAGGCTGAACCGTGTACCCTCCCTTAGCAGGCAACCTAGCCCA
TGCAGGAGCCTCAGTAGATCTAACCATCTTCTCCTTACACTTGGCAGGAGTTTCCTCTATTTTAGGAGCCATCA
ACTTCATCACAACAATTATCAACATAAAGCCCCCCGCAATGTCACAGTACCAAACCCCTCTCTTCGTATGATC
CGTAATAATTACCGCCGTACTATTACTCCTCTCACTTCCTGTGCTAGCAGCCGGTATTACAATGCTGCTAACAG
ATCGGAACCTAAATACAACCTTCTTCGACCCAGCAGGAGGAGGAGACCCCATCTTATACCAACACTTATTC
>Z045_2
TACCCTTTATCTACTATTTGGAGCCTGGGCCGGTATAGTAGGAACAGCTCTAAGCCTTCTAATTCGCGCTGAAT
TAGGCCAACCCGGAACCCTGCTCGGAGACGACCAAATCTACAACGTAGTTGTAACCGCACACGCATTTGTAA
TAATCTTCTTTATAGTAATGCCAATTATAATTGGAGGATTCGGTAACTGACTTGTTCCTCTAATAATTGGCGCT
CCCGACATGGCATTCCCCCGAATAAACAATATAAGCTTCTGACTCCTCCCTCCCTCATTTCTACTACTCCTCGC
ATCCTCTATAGTTGAAGCTGGAGCAGGAACAGGCTGAACCGTGTACCCTCCCTTAGCAGGCAACCTAGCCCA
TGCAGGAGCCTCAGTAGATCTAACCATCTTCTCCTTACACTTGGCAGGAGTTTCCTCTATTTTAGGAGCCATCA
ACTTCATCACAACAATTATCAACATAAAGCCCCCCGCAATGTCACAGTACCAAACCCCTCTCTTCGTATGATC
CGTAATAATTACCGCCGTACTATTACTCCTCTCACTTCCTGTGCTAGCAGCCGGTATTACAATGCTGCTAACAG
ATCGGAACCTAAATACAACCTTCTTCGACCCAGCAGGAGGAGGAGACCCCATCTTATACCAACACTTATTC
